# *Sideritis raeseri*—Modified Coatings on Ti-6Al-4V as a Carrier for Controlled Delivery Systems of Active Substances

**DOI:** 10.3390/ma17102250

**Published:** 2024-05-10

**Authors:** Karina Niziołek, Dagmara Słota, Julia Sadlik, Edyta Kosińska, Klaudia Korzeń, Josef Jampilek, Agnieszka Sobczak-Kupiec

**Affiliations:** 1 Department of Materials Science, Faculty of Materials Engineering and Physics, Cracow University of Technology, 37 Jana Pawła II Av., 31-864 Krakow, Poland; 2 Department of Analytical Chemistry, Faculty of Natural Sciences, Comenius University, Ilkovicova 6, 842 15 Bratislava, Slovakia; 3 Department of Chemical Biology, Faculty of Science, Palacky University Olomouc, Slechtitelu 27, 783 71 Olomouc, Czech Republic

**Keywords:** flavonoids, drug delivery system, polymers, ceramics, metallic coatings, composite

## Abstract

The search for the ideal metallic material for an implant is still a difficult challenge for scientists due to the phenomenon of corrosion and the consequent disruption of the implant structure. Prevention is the application of coatings that protect the implant, activate the tissues for faster regeneration, and also prevent inflammation through antibacterial and antiviral effects. The present study focuses on the selection of components for a Ti-6Al-4V alloy coating. These days, researchers are taking an intense interest in extracts of natural origin. It was decided to take a look at *Sideritis raeseri*, which contains vitamins and valuable elements and is rich in polyphenols, as well as antioxidants. The composition of coatings based on a PEG polymer reinforced with brushite and the *S. raeseri* extract with the proteins L-carnosine, fibroin, or sericin was developed. The samples were subjected to detailed physiochemical analysis, including potentiometry and electrical conductivity analysis, Fourier transform infrared spectroscopy (FT-IR), scanning electron microscopy (SEM), X-ray diffraction (XRD) analysis, and UV-VIS spectroscopy. The study demonstrated that polyphenols were successfully released from the coatings during incubation in vitro. The osteointegration process can be supported by a number of factors, such as the release of polyphenols from implant coatings to prevent bacterial, viral, and fungal infections. Subjecting the samples to 14 days of incubation demonstrated their interactions with the incubation fluids, an ion exchange between the medium and the materials. An analysis of the surface morphology exhibited the presence of brushite crystals and their increased number after incubation, indicating the bioactivity of the formed coatings.

## 1. Introduction

The development of civilization, the accelerated pace of life, and the increase in pollution nowadays often lead to the development of diseases in society. It has been noted that the incidence of orthopedic disease problems is not improving despite significant medical advances. Today’s orthopedic surgery is confronted with a wide range of conditions, which not infrequently lead to replacement and regeneration to treat bone defects [1].

For the aforementioned applications, such as bone implants, three types of metal are used: stainless steel, cobalt–chromium alloy, as well as titanium and its alloys. Due to their superior properties, titanium alloys have displaced the other biomaterials and have become the most widely used materials in implantology [2,3]. Titanium and its alloys are characterized by a number of features that make them ideal for both surgical and dental implantology, such as high biocompatibility, a high load-bearing capacity, corrosion resistance, and high hardness and wear resistance [4]. The most common metal implant material is titanium alloy Ti-6Al-4V, which is a two-phase alloy. It consists of phase α, which is stabilized with aluminum, and phase β, which is stabilized with vanadium. The titanium alloy discussed here is known for its low density and high Young’s modulus. Ti-6Al-4V exhibits significant corrosion resistance, both in air and in physiological fluids. It is important to note that, due to its much greater strength than pure titanium, it is more commonly used in implantology. Ti-6Al-4V is also characterized by its non-toxicity, which translates into its non-rejection in the human body. It can remain in the living body for up to 20 years. It should be noted that titanium alloys possess the capability to fuse with bone and also have a low modulus of elasticity that matches that of bone. The Ti-6Al-4V alloy reduces bone degradation and protects against fractures around the prosthesis by evenly distributing loads between bones and implants [5]. 

Calcium phosphates such as hydroxyapatite (HA), tricalcium phosphate (TCP), and biphasic calcium phosphates are the best known and most commonly used in bone implantology. Calcium phosphates can be used in the treatment of bone defects, biomedical cement, or implant coatings, among other applications [6]. Brushite (CaHPO_4_∙2H_2_O, DCPD) is a calcium hydrogen phosphate dihydrate belonging to calcium phosphate-based ceramic biomaterials. The structure of brushite is characterized by parallel layers of calcium with hydrogen phosphate groups placed between metal ions. Water molecules are placed between layers of calcium phosphate [7]. The compound under investigation is present in human bones, and it was initially discovered in a bat. DCPD particles can be found in kidney stones, bones, or non-collagenous organic matter surrounding non-mineralized, ordered collagen fibers [8]. These particles can be used as a storage material for calcium ions and phosphate ions. DCPD has the unique characteristic of rapid formation and decomposition. This property may contribute to the formation of kidney stones, but it can also have a positive effect on the synthesis of bone cements. Research has demonstrated that brushite possesses properties that are relevant to bone implants. The compound is known for its biocompatibility and bioresorbability, as well as its osteoconductive and osteoinductive qualities. The compound is rapidly absorbed in body fluids. This has the effect of significantly shortening the process of new bone formation. These characteristics mean that DCPD can be successfully used as a bone replacement material or as a bioactive layer on the surface of titanium implants. It is important to note that it has higher solubility and lower surface energy compared to HA, making it a more desirable compound for biomedical applications than HA [9]. 

Osteointegration occurs when the bone and implant come into contact, and this process can be influenced by the presence of DCPD. Osteointegration occurs by inducing osteoblast activity. The rapid rate of resorption of the brushite affects the appearance of a gap between the bone and the bone cement. The resulting gap allows bone remodeling in subsequent stages of the process. The rate of brushite resorption slows down due to its transformation into hydroxyapatite. New bone material forms in the gaps between the bone cement and the bone. Brushite’s involvement in osteosynthesis leads to greater bone regeneration compared to hydroxyapatite [9,10,11]. 

Proteins are heteropolymers composed of 20 different amino acids that are linked together via peptide bonds in various sequences. This structural diversity enables a broad range of reactions and interactions to take place. Proteins serve structural, transport, and regulatory functions in all living organisms [12]. L-carnosine is a protein with a building function in vertebrates, including humans. L-carnosine is a dipeptide that consists of β-alanine and L-histidine. This compound is naturally found in the greatest amount in muscles, where it is responsible for the functional activity of muscles. This protein can also be found in high concentrations in brain tissue, for which it is an antioxidant and antiglycosite. The presence of L-carnosine accelerates the scavenging of free radicals. Through a combination of free radical neutralization and metal chelation, it contributes to the inhibition of lipid oxidation [13,14]. L-carnosine protects the body from the harmful effects of metal ions such as Cu, Co, and Cd by forming complexes with them. Additionally, it binds to glucose, providing protection against excess glucose [14]. Fibroin is a protein found in silk fibers that serves a structural role. The fibroin protein is composed of two chains, a light chain (L-fibroin) and a heavy chain (H-fibroin), which are connected via disulfide bonds. Additionally, fibrohexamerin is linked to the previous two chains via a non-conjugal bond [15]. The protein is composed of a repeating sequence of alanine, glycine, and serine amino acids [16]. Fibroin has no adverse reactions to the living organism, and its implanted fibers degrade after approximately six months. The protein is used in tissue engineering and hydrogel production due to its high strength and biocompatibility [17]. Sericin is a protein composed of amino acids, including serine, glycine, glutamic acid, and tyrosine [18]. Its antibacterial effect has been confirmed by inhibiting the growth of bacterial colonies when treated with sericin. Sericin is extracted from silk during processing. It possesses bioactive, moisturizing, and antioxidant properties. Due to these characteristics, it is commonly used in regenerative medicine, particularly for skin recontouring, and as a drug transporter [19,20]. 

Flavonoids are a group of polyphenolic compounds commonly found in nature. Flavonoids are distinguished by characteristics such as biological activity and antioxidant activity. This clearly indicates their ability to scavenge free radicals, as well as chelate metal ions. They are divided into numerous subclasses, including flavonols, flavanonols, flavones, isoflavones, flavones, and anthocyanins. Flavonoids contain a basic C6-C3-C6 structure, i.e., two phenolic rings connected to a C3 ring characterized by different degrees of oxidation. Approximately 4000 flavonoids have, so far, been recognized in the natal environment [21,22,23,24,25]. Two of the most popular antioxidants are kaempferol and quercetin, which are flavones. Their synthesis is closely linked to sunlight, and they are most abundant in the skins of fruits and leaves. Isoflavones have a significant protective effect on reducing “bad” LDL cholesterol in relation to HDL. They also demonstrate a reduction in carcinogenic processes by disrupting oxygen delivery mechanisms and scavenging free radicals [26,27].

*Sideritis reaseri* is a plant that belongs to the genus *Sideritis*, and the name derives from the Greek word “sideros”, which means iron, as it was used in ancient times to treat wounds from iron weapons. The most common name for this plant is mountain tea [28].

The plant is known for its anti-inflammatory and tonic properties [29]. It owes its valuable qualities to its content of polyphenols, flavonoid glycosides, and also hydroxyflavones such as hypoaleptin [30]. The plant is used as a tea for the treatment of inflammation and stomach disorders, and it is also used as an ingredient in dietary supplements for anemia [31]. 

It is possible to create a system that releases active substances using biodegradable materials and bioactive ceramics. The aim of the present study was to develop such coatings for metal alloys, particularly the Ti-6Al-4V alloy. Careful attention was paid to the selection of natural ingredients, which is why a plant extract was used in this work. The compositions are based on the polymer polyethylene glycol (PEG), the ceramic phase brushite, extract from the polyphenol-rich plant *S. reaseri*, and the protein L-carnosine, fibroin, or sericin. Such a composite coating guarantees better properties, as it combines the characteristics of polymeric and ceramic materials. The materials in question were subjected to a detailed physicochemical analysis to select the best composition for coating metallic biomaterials. The composites were incubated under conditions simulating a living organism in selected incubation fluids, followed by pH-metric and conductometric analysis. The kinetics of the release of polyphenols from the coatings was determined using the Folin–Ciocâlteu method. The surface morphology was checked using scanning electron microscopy (SEM). The developed composition showed great potential as a material for regenerative medicine applications.

## 2. Materials and Methods

### 2.1. Reagents

Reagents for ceramic synthesis, i.e., disodium hydrogen phosphate dihydrate (Na_2_HPO_4_∙2H_2_O), calcium nitrate tetrahydrate (Ca(NO_3_)_2_∙4H_2_O), and ammonia water (NH_4_OH, 25%), were purchased from Sigma-Aldrich (Darmstadt, Germany). Dried *Sideritis raeseri* leaves were purchased from Natur-Vit (Pińczów, Poland). For the synthesis of the coating materials, L-carnosine, fibroin, sericin, poly(ethylene glycol) Mn 10000 (PEG), poly(ethylene glycol) diacrylate average Mn 700 (PEGDA), and 2-hydroxy-2-methylpropinophenone 97% were purchased from Sigma-Aldrich (Darmstadt, Germany). Ringer’s solution and artificial saliva were prepared using NaCl, KCl, and CaCl_2_ from Eurochem BGD (Tarnów, Poland), as well as from Na_2_HPO_4_∙H_2_O, Na_2_S∙9H_2_O from Chempur (Piekary Śląskie, Poland) and urea from Stanlab (Lublin, Poland). Phosphate-buffered saline tablets were purchased from OXOID (Basingstoke, UK). The antioxidant assays used were gallic acid, 2,2-Diphenyl-1-picrylhydrazyl (DPPH), from Sigma-Aldrich (Darmstadt, Germany) and Folin–Ciocâlteu’s reagent, sodium carbonate (Na_2_CO_3_), and ethanol 96% from Chempur (Piekary Śląskie, Poland). A Hydrolab-model HLP 5sp (Wodzisław Śląski, Poland) unit was used to obtain demineralized water for all solutions.

### 2.2. Preparation of Brushite

Brushite powder was synthesized using the wet precipitation method from Na_2_HPO_4_∙2H_2_O and Ca(NO_3_)_2_∙4H_2_O salts [32]. Into 250 mL of a Na_2_HPO_4_∙ 2H_2_O solution of 0.5 mol/L was placed, on a magnetic stirrer, 250 mL of a Ca(NO_3_)_2_∙4H_2_O solution of 0.5 mol/L, which was dropped at a rate of 1 drop per second. During synthesis, the pH of the solution was monitored and maintained in the range of 6–6.5 with 25% ammonia water. After infusion, the brushite was left to age the precipitate for 24 h. After this time, the precipitate was washed with distilled water to a neutral pH and left to dry in a POL-EKO laboratory dryer model SLW 400 (Wodzisław Śląski, Poland) at 104 °C for 4 h. The brushite was then subjected to roasting in an oven at 1000 °C for 2 h.

### 2.3. X-ray Diffraction Analysis

To conduct a structural characterization of the resulting ceramic powder after wet synthesis at zero time, X-ray diffraction analysis was conducted using a Malvern Panalytical Aeris X-ray diffractometer equipped with a PIXcel1D-Medipix3 detector (Malvern Panalytical, Malvern, UK) and Cu Kα radiation. Measurements were performed with a step size of 0.0027166° 2θ across a 2θ range of 10–60°, with each step taking 340.425 s.

### 2.4. Preparation of Sideritis raeseri Extract

In order to prepare the *S. raeseri* extract, an aqueous extraction process was carried out in a Soxhlet apparatus. A cellulose extraction thimble was loaded with 5 g of dried *S. raeseri*, and 200 mL of distilled water was added to a round-bottomed flask. The resulting system was then brought to a boiling point, and the entire process was carried out for 12 h.

### 2.5. Preparation of Coatings

For the preparation of coatings, a 15% PEG solution was made by dissolving the polymer in an aqueous extract of *S. raeseri*, obtained as described in Section 2.4. The PEG solution was mixed in a 1:1 ratio with an aqueous solution of 2% L-carnosine, fibroin, or sericin. The composition was reinforced with the addition of brushite obtained via the wet precipitation method, as previously described in Section 2.2. The mass of brushite constituted 15% relative to the dry weight of the other components. The mixture was then combined with 50 µL of 2-hydroxy-2-methylpropinophenone and 2 mL of PEGDA with a molecular weight of 700. In the next step, 1 mL of the mixture was applied to ethanol-purified Ti-6Al-4V plates measuring 2 cm × 3 cm to form a thin film, which was then photocrosslinked under UV light using a Medilux UV 436 HF lamp (Medilux, Korntal-Münchingen, Germany) for 4 min. Table 1 presents the composition of the coatings. During sample synthesis, larger blends of components were prepared, from which a volume of 1 mL per coating was subsequently taken.

### 2.6. Fourier-Transform Infrared Spectroscopy Analysis 

In the present study, the individual functional groups in the obtained coatings were identified using Fourier transform infrared spectroscopy (FT-IR). The analysis was carried out using a Nicolet iS5 FT-IR spectrometer equipped with an iD7 ATR accessory (Thermo Scientific, Loughborough, UK) in the range from 4000 to 400 cm^−1^ (32 scans at 4.0 cm^−1^ resolution) under room conditions.

### 2.7. Incubation In Vitro

#### 2.7.1. pH-Metric and Conductometric Analysis

In order to evaluate the behavior of the materials in an environment simulating the conditions of a living organism, the coatings were subjected to incubation tests in an artificial biological medium. Four fluids were selected, i.e., distilled water, PBS, Ringer’s fluid, and artificial saliva. PBS was obtained by dissolving 1 tablet in 100 mL of distilled water, while the other two fluids were obtained as described previously [33]. The coatings were placed in 100 mL of each liquid and sealed in airtight, sterile containers. Incubation was carried out for 14 days at 36.6 °C in a POL-EKO incubator, model ST 5 B SMART. The pH value and ionic conductivity measurements were determined after 1, 3, 7, 9, and 14 days using an Elmetron CX-701 pH-meter (Zabrze, Poland).

#### 2.7.2. Determination of Sorption Capacity of Biomaterials

During the incubation period, in the fluids and conditions described in detail in Section 2.7.1, the sorption capacity of the coatings was also analyzed. For this purpose, the mass of a dry sample was recorded in detail, and then the sample was placed in liquid for the next step. After 30 min, the coating was removed, and the excess liquid from the surface was gently collected with filter paper and weighed. After the weight was recorded, the sample was again placed in the liquid. This activity was repeated after 1 h and 1, 3, 7, 9, and 14 days for all samples in all liquids. The swelling ability was determined analogously to those described previously [34].

### 2.8. Determination of Total Polyphenol Content

The Folin–Ciocâlteu colorimetric assay was used to determine the total polyphenol content (TPC) of the extracts tested. For this purpose, a 5 mg/mL gallic acid solution and a supersaturated Na_2_CO_3_ solution were prepared. To create the calibration curve, a series of dilutions of the gallic acid working solution was prepared at the following concentrations: 0.05, 0.15, 0.25, 0.35, and 0.5 mg/mL. The resulting 20 μL of calibration standards, 1.6 mL of distilled water, and 100 μL of Folin–Ciocâlteu reagent were placed in a cuvette. After 3 min, 300 μL of saturated Na_2_CO_3_ solution was added to the mixture. The subsequently prepared samples were thoroughly mixed with a pipette and thermostated for 30 min at 40 °C.

*S. raeseri* extract samples were prepared in an identical manner and diluted ten times. The gallic acid solution was replaced with 20 μL of *S. raeseri* extract. Measurements were carried out at 765 nm on a Genesys 180 UV-Vis spectrophotometer from Thermo Scientific (Loughborough, UK). The method has previously been described in detail [35].

### 2.9. Determination of Antioxidation Potential via DPPH Method

To test the antioxidant efficacy of the extract obtained from *S. raeseri* during aqueous extraction in a Soxhlet apparatus, the ability to inactivate free radicals was tested using 2,2-diphenyl-1-picrylhydrazyl (DPPH). A solution of DPPH was prepared by dissolving 19.71 mg of this compound in 100 mL of 96% ethanol. The resulting solution was diluted to an absorbance value of approximately 0.9. Control sample A_0_ consisted of 2 mL of the prepared DPPH solution and 60 μL of 96% ethanol. Absorbance was measured on a Genesys 180 UV-Vis spectrophotometer from Thermo Scientific (Loughborough, UK) at a wavelength of 517 nm [35].

*S. raeseri* extract samples were measured in an identical manner, previously diluted two times. Then, 60 μL of ethanol was replaced with the obtained aqueous extract. Absorbance measurements were taken after approximately 10 min at room temperature at the same wavelength as that used for the control sample. Three replicates each were performed for the test sample. The measurements were averaged (*A*), and the antioxidant capacity of the test extract was calculated according to the following formula:

(1) $$\%\;inhibition=\frac{A_{0}-A}{A_{0}}\cdot 100$$

### 2.10. Determination of Release Kinetics of Polyphenols

In order to study the amount of active ingredient released from the obtained composite coatings, an Electrolab EDT-08lx release water bath (Mumbai, India) was used. Samples of the obtained coating materials were placed in baskets in a water bath of 100 mL of distilled water. Stirring was then switched on at 100 rpm at 36.6 °C. After the specified time, 1 mL of solution was taken from each station, and 1 mL of distilled water was added to the system. Samples were taken at the following intervals: 60 min, 180 min, 1 day, 5 days, and 7 days. For the determination of the total polyphenol content (TPC), the samples obtained were analyzed using the Folin–Ciocâlteu method in a procedure identical to that described in Section 2.8.

### 2.11. Morphology Analysis 

The surface, internal structure, deformation, and chemical composition of objects can be observed and evaluated by examining a material with a scanning electron microscope (SEM) [36]. Samples were examined before and after being incubated in fluids for 2 weeks to visualize their surface morphology. The measurements were conducted using a scanning electron microscope (SEM) JEOL 5510LV (Tokyo, Japan). Before the SEM measurement, the composites were pre-dried and coated with a gold nanolayer using a vacuum sputter coater Cressington model 108 auto (Watford, UK).

## 3. Results

### 3.1. X-ray Diffraction Analysis 

In order to study the crystal structure of the synthesized ceramic powder, an X-ray diffraction (XRD) study was performed.

**Figure 1:**
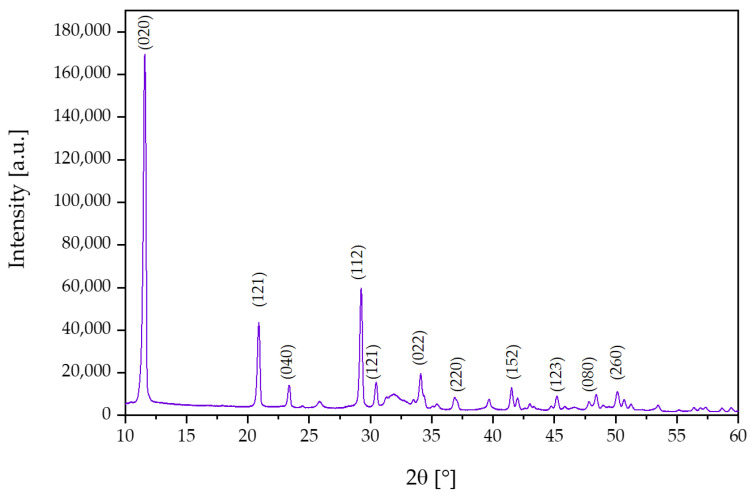
X-ray diffraction (XRD) spectrum for synthesized brushite.

The obtained reflections for the test sample in the XRD study are presented in Figure 1. The XRD analysis revealed characteristic peaks assigned to the single-stranded brushite structure that corresponded to space group Ia. Moreover, the analysis of the obtained diffractogram, according to file card No. 00-011-0293 in the ICDD database, indicates characteristic peaks at two theta equal to 11.58° (020), 20.86° (121), 23.35° (040), and 29.21 (112) [7,37,38,39].

### 3.2. Fourier-Transform Infrared Spectroscopy Analysis

**Figure 2:**
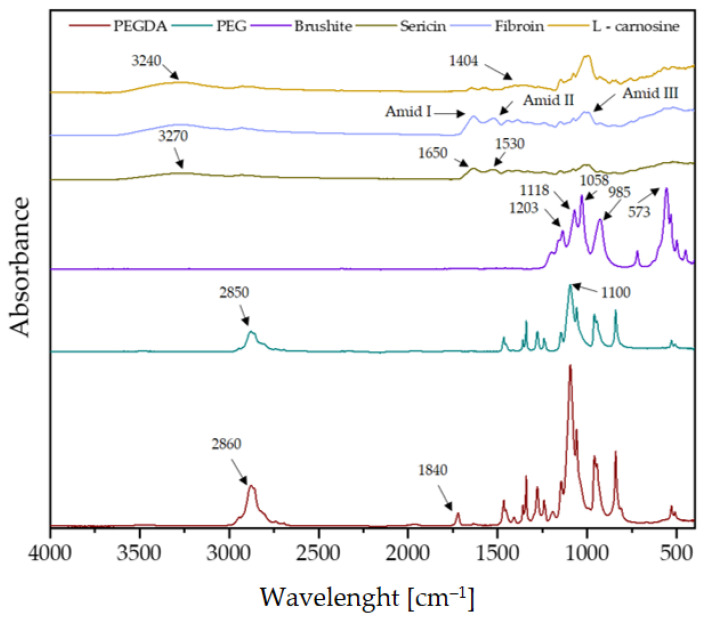
FT-IR spectra for the pure components included in the coatings.

FT-IR spectrometric analysis was used to determine the chemical composition of the synthesized coatings. Figure 2 shows the spectra of the pure coating components. FT-IR spectrometric analysis makes it possible to show the characteristic spectra corresponding to the stretching and bending vibrations of functional groups. The crosslinking agent PEGDA 700 is characterized by an absorption band at 2860 cm^−1^, which is responsible for C-H stretching vibrations [40]. The spectrum of the PEG polymer is characterized by distinct peaks in the region of 2850 cm^−1^ derived from the ethylene group -CH_2_. 

Based on the brushite spectrum, phosphate groups occurring at wavelengths between 550 cm^−1^ and 1030 cm^−1^ were identified. The peaks at 573 cm^−1^, 985 cm^−1^, and 1058 cm^−1^ correspond to the PO_4_^3−^ group. The FT-IR spectrum for the brushite shows 1118 and 1203 cm^−1^ characteristic peaks attributable to the monohydrogen phosphate ion HPO_4_^2−^ [41].

A broad band of amide A (3270 cm^−1^) is visible for sericin. Also characteristic of sericin are such peaks as 1650 cm^−1^ and 1530 cm^−1^, corresponding to amide I and amide II. A peak at 1400 cm^−1^ can also be seen. The presence of increased absorbance at the wavelengths shown is related to the high content of alkylhydroxy carboxylic acids found in the sericin molecule [42].

The spectrometric spectrum of fibroin demonstrates characteristic groups of vibrations, the so-called amide modes. The band belonging to amide I occurs in the 1700–1590 cm^−1^ region and characterizes C=O stretching vibrations. On the other hand, the 1590–1460 cm^−1^ region is amide II, which is characterized by N-H and C-N bending vibrations. Amide III in the 1190–1280 cm^−1^ area corresponds to N-H bending and C-N stretching vibrations [43].

The L-carnosine spectrum is indicated with a yellow line, and it depicts -NH_2_ stretching vibrations at about 3240 cm^−1^. Values of 1568 cm^−1^ and 1404 cm^−1^ illustrate asymmetric and symmetric carboxylate (COO-) stretching vibrations, respectively. In turn, the peaks at 2850 and 2785 cm^−1^ characterize the symmetric and asymmetric stretching vibrations of C-H groups, respectively [44,45].

**Figure 3:**
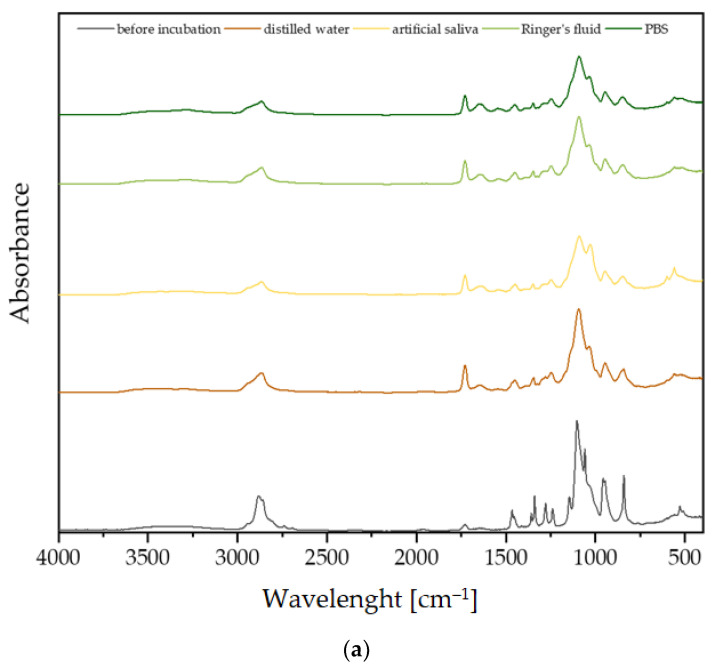

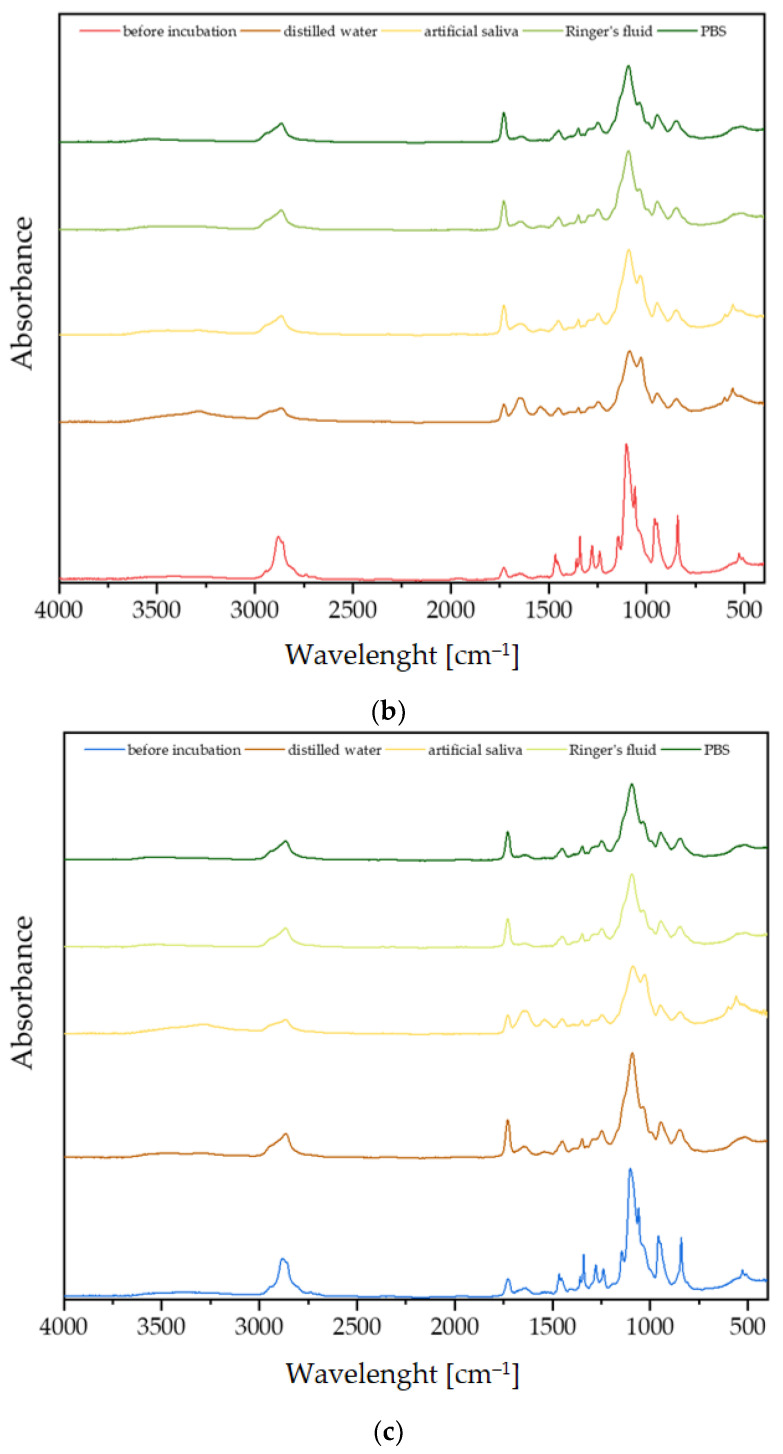
FT-IR spectra of the formed coatings before incubation and after incubation in distilled water, artificial saliva, Ringer’s fluid, and PBS for samples with (**a**) l-carnosine, (**b**) fibroin, and (**c**) sericin.

FT-IR analysis was performed on samples containing sericin, fibroin, and L-carnosine, and the spectra are presented in Figure 3. Spectrometric analysis was performed before and after incubation. The peaks characteristic of the PEGDA 700 crosslinker, the PEG polymer, and the brushite were observed in the spectra of all coatings before incubation. It should be noted that the absorption spectrum for fibroin and sericin are very similar. This means that these compounds have a similar chemical structure and have similar functional groups. For this reason, it is very difficult to clearly identify materials containing these two compounds.

Based on the spectra obtained, it can be concluded that all synthesized coatings behave similarly in incubation fluids. There is a noticeable weakening of the signal from the crosslinking agent PEGDA and from PEG at 2850 cm^−1^ in all physiological fluids. The peak at 1840 cm^−1^, coming from PEGDA, showed an increase in absorbance. This relationship was repeatable in all incubation fluids. This observation suggests the degradation of the polymer occurring after incubation in physiological fluids. These observations indicate that the materials are incubated very similarly in SBF, distilled water, artificial saliva, Ringer’s fluid, and PBS solution.

### 3.3. Incubation In Vitro

#### 3.3.1. pH-Metric Analysis

**Figure 4:**
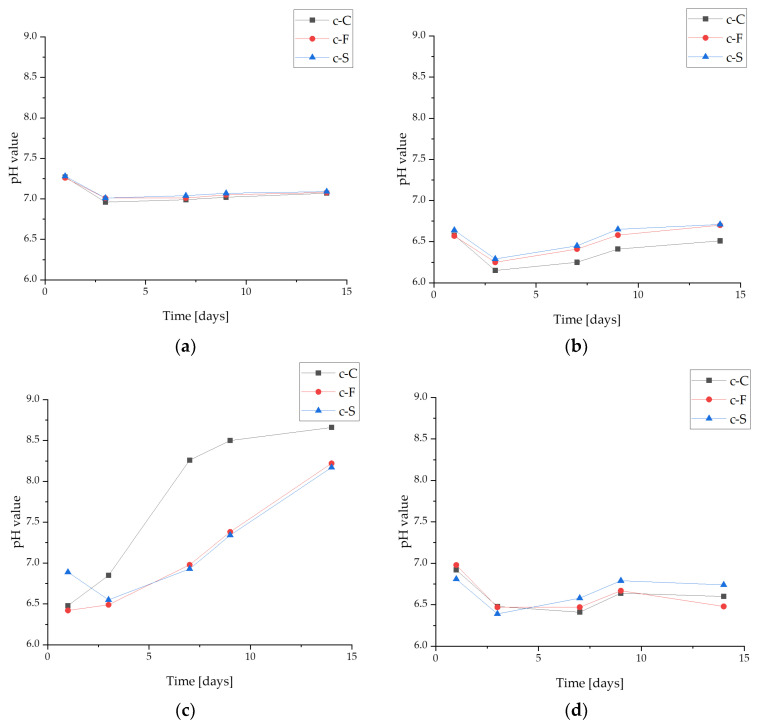
pH-metric analysis of the coatings during a 14-day incubation in fluids simulating the biological environment: (**a**) incubation in PBS; (**b**) incubation in Ringer’s fluid; (**c**) incubation in artificial saliva; and (**d**) incubation in distilled water.

Potentiometric measurements, including the observation of a change in the pH value, make it possible to determine the stability of the biomaterial in a given environment. This study was conducted with four fluids differing in their composition and initial reactions. For a better comparison, all values were compiled in the same range for pH from 6 to 9 (Figure 4). It is clearly noticeable that the pH changed the least during incubation in PBS, a fluid that is characterized by buffering properties. This fluid corresponds in composition to saline. In Ringer’s fluid and water, the materials behave similarly. However, in the case of the first fluid, the pH changes are probably a result of an interaction with solution ions. In distilled water, there are no ions, so the changes may be due to the leaching of components from the coating, which would explain the lack of a linear relationship in this fluid. The largest changes were observed in the artificial saliva, and here, too, partial degradation of the polymer matrix was observed and, thus, chipping of ceramic grains to the bottom of the measuring vessel. Significantly, the lesions may also result from the release of *S. raeseri* into the incubation medium. In PBS, due to its buffering properties, this change is not clearly noticeable, but the release of flavonoids into the fluid would also explain the changes occurring in water.

It should also be emphasized that no effect of material composition on pH values was observed. Coatings containing L-carnosine and fibroin, as well as sericin, behaved similarly in all fluids.

#### 3.3.2. Conductivity Analysis

**Figure 5:**
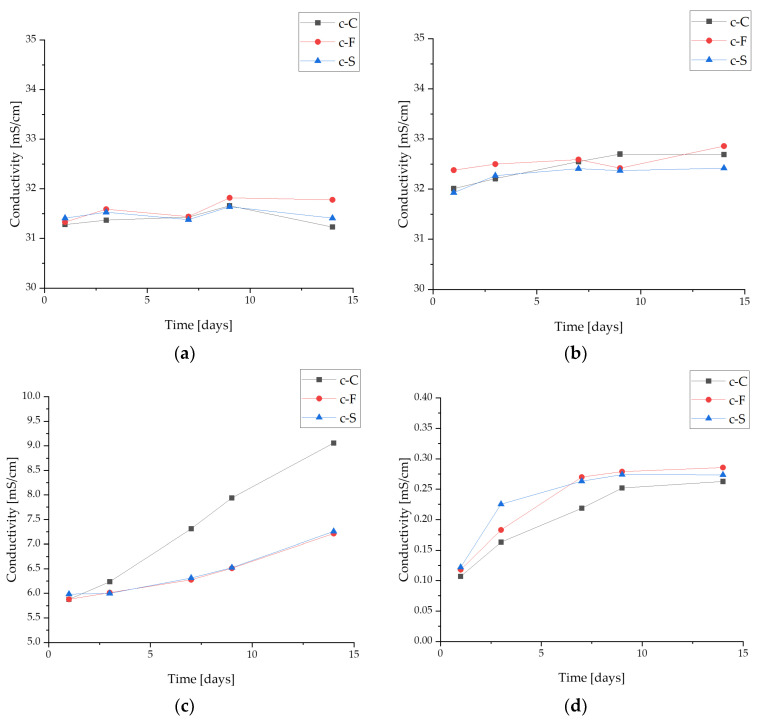
Conductometry analysis of the coatings during a 14-day incubation in fluids simulating the biological environment: (**a**) incubation in PBS; (**b**) incubation in Ringer’s fluid; (**c**) incubation in artificial saliva; and (**d**) incubation in distilled water.

Studying the electrolytic conductivity of reacting compounds or the resulting products enables the analysis of ionic compounds and the monitoring of chemical reactions by obtaining conductivity measurements. Conductivity measurements were made in four fluids simulating the body’s internal environment. The results of conductivity changes during incubation are shown in Figure 5.

Changes in conductivity values indicate an ion exchange between the test material and the corresponding incubation fluid. The largest noticeable changes in conductivity were noted for the c-C sample after a 14-day incubation in artificial saliva. The observed relationship may be due to the progressive degradation of the coating during incubation and interaction with the ions of the fluid solution simulating the internal environment of the organism. These changes may also be due to the release of *S. raeseri* extracts from the polymer matrix and calcium phosphate ceramics, which are part of the coatings. During incubation in the PBS buffer solution and Ringer’s fluid, no major changes in ionic conductivity were observed. Moreover, in distilled water, there is a release of ions from the resulting coatings; however, the changes are minimal compared to samples measured in artificial saliva.

#### 3.3.3. Determination of Sorption Capacity of Biomaterials

**Figure 6:**
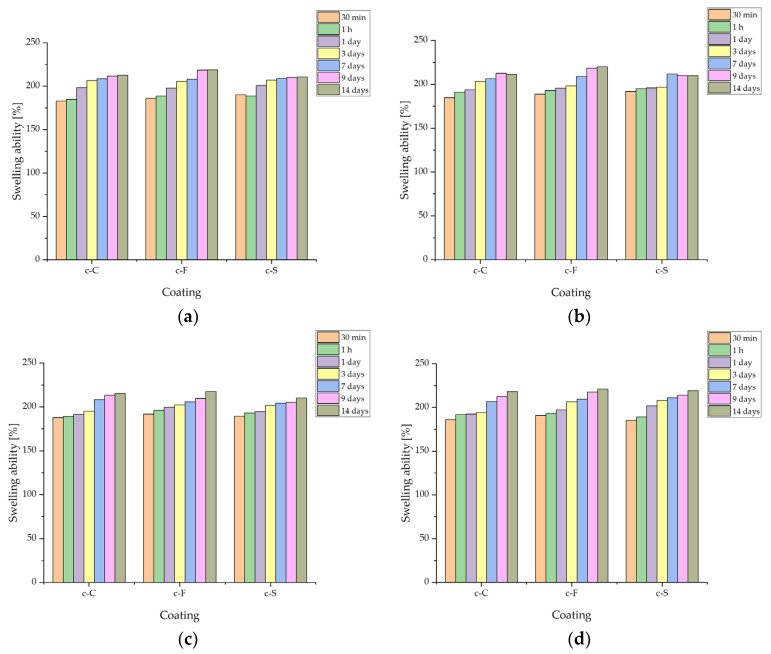
Sorption capacity of the coatings during a 14-day incubation in fluids simulating the biological environment: (**a**) incubation in PBS; (**b**) incubation in Ringer’s fluid; (**c**) incubation in artificial saliva; and (**d**) incubation in distilled water.

During the evaluation of sorption capacity (Figure 6), the swelling ability of composite coatings poured onto Ti-6Al-4V plates was evaluated. Swelling consists of the ability of the liquid medium to penetrate into the structure of the material, filling the free spaces between the polymer chains. Based on previous studies, it can be concluded that the swelling ability is inhibited somewhat due to the presence of ceramics; however, a swelling parameter of more than 200% after 14 days was observed in all samples [34,35]. The highest increase was observed at the initial stage of incubation, i.e., after 30 and 60 min. After that, the value slowly stabilized. No significant difference was observed between fibroin, sericin, and L-carnosine; in all liquids, it was fibroin that obtained the highest values, but the observed differences were on the order of less than 10% compared to the other coatings in the same liquid. The tendency for the lowest values was observed for fibroin-containing biomaterials.

Significantly, the material’s sorption capacity and its swelling ability can be used for the targeted therapy and transport of active ingredients like drugs. The elution of an ingredient from the interior of a biomaterial is one of the strategies of modern therapies, based on the delivery of biomolecules that provide a therapeutic effect directly to the diseased site [46]. 

### 3.4. Determination of Total Polyphenol Content

The Folin–Ciocâlteu method was used to determine the total polyphenol content (TPC). The Folin–Ciocâlteu reagent contains a mixture of phosphomolybdic and phosphotungstic acids that, when reduced, produce a blue chromophore with maximum absorption at 765 nm. This test is based on an electron transfer reaction between the F-C reagent, which is the oxidant, and the antioxidant, the electron donor. The degree to which the reagent changes color after electron extraction depends on the reducing activity of the antioxidant compounds [47,48]. 

The total polyphenol content (TPC) for the *S. raeseri* extract was expressed as a gallic acid equivalent (mg GAE/g) and was 31.64 ± 0.69 mg GAE/g.

### 3.5. Determination of Antioxidation Potential via DPPH Method

To evaluate the antioxidant potential of the *S. raeseri* extract, the DPPH method was used. The procedure of the method is based on the reduction of 2,2-diphenyl-1-picrylhydrazyl (DPPH) free radicals dissolved in ethanol with antioxidant substances containing hydrogen donor groups. Electron delocalization results in the appearance of a dark purple color in the molecule and maximum absorption of the ethanol solution at 517 nm [49,50]. 

The antioxidant efficiency of the extract obtained during aqueous extraction from the plant *S. raeseri* was expressed as percentage inhibition, which reached 84.19 ± 0.71%.

### 3.6. Determination of Release Kinetics of Polyphenols

**Figure 7:**
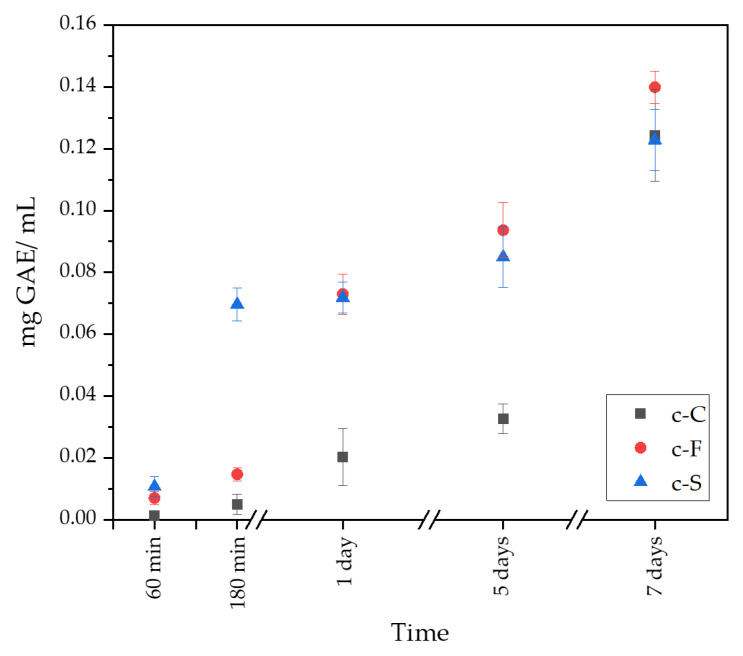
Release profile of *Sideritis raeseri* extract from coatings.

In the obtained ceramic–polymer coatings, an equal amount of active substances, which were polyphenols derived from *S. raeseri* extract, were placed at the synthesis level. Figure 7 shows the release profile of polyphenols from the obtained coatings. The release profile shows the difference between the number of polyphenols released as a function of time. After 60 min, a small number of polyphenols was released from the c-F and c-S coating, but from the c-C sample, TPC was not determinable. However, after 180 min, the sample containing sericin reached about four times the polyphenols in relation to the c-C and c-F samples. The c-C sample released a lower polyphenol content compared to the other coatings by day 5 of the test. After 7 days of incubation, the c-C and c-S coatings released similar amounts of TPC, while the sample containing fibroin released the most polyphenols. The release profiles obtained are consistent with the results achieved when testing the sorption capacity of the obtained coatings. As the swelling ratio increases, the amount of released polyphenols from the obtained ceramic–polymer coatings grows. It is possible to conclude that the addition of fibroin could increase the amount of released active ingredient. Moreover, the process of releasing polyphenols from the coatings proceeded gradually, without sudden peaks.

### 3.7. Morphology Analysis

**Figure 8:**
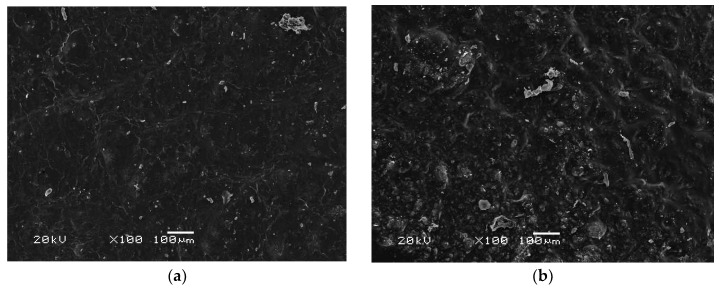

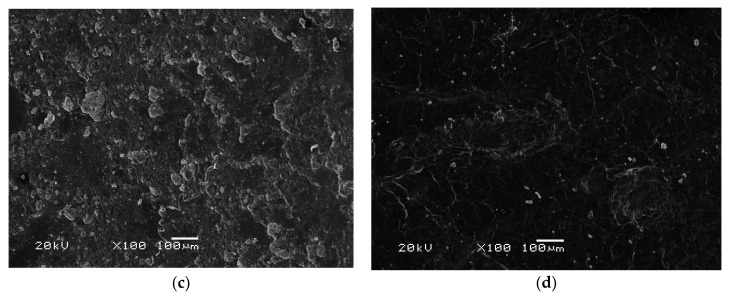
Analysis of the surface morphology of the sample with fibroin c-F (**a**) before incubation, (**b**) after incubation in Ringer’s fluid, (**c**) after incubation in PBS, and (**d**) after incubation in distilled water.

The composite sample c-F was analyzed using scanning electron microscopy both before and after a 14-day incubation in Ringer’s liquid, PBS, and distilled water. These incubations were carried out under conditions that simulate an organism’s environment at a temperature of 36.6 °C (Figure 8). The matrix was observed to have brushite crystals before incubation. The ceramic phase was evenly distributed, with crystals of a similar size and no visible agglomerations. After the incubation, newly formed crystals of brushite were noticeable, with most of them formed in Ringer’s liquid and PBS. This crystal growth could be attributed to the presence of calcium in the incubation solutions. Samples incubated in water showed no significant changes, with the number of brushite crystals being similar in samples before and after incubation. The aqueous environment does not have a significant effect on brushite growth due to the absence of other ions in the solution. Notably, it does not adversely affect the coating, as there are no signs of degradation or deformation of the material.

## 4. Discussion

In conclusion, the obtained ceramic–polymer coatings on a Ti-6Al-4V alloy modified with *S. raeseri* extract were thoroughly characterized. As a result of the proposed wet precipitation method for obtaining calcium phosphate ceramics, a brushite phase with high crystallinity was obtained, as confirmed via XRD spectrum analysis. Moreover, the analysis of T-IR spectrum demonstrated characteristic peaks for brushite for the HPO_4_^2−^ and PO_4_^3−^ functional groups. Moreover, FT-IR analysis of the obtained composites indicates the presence of characteristic functional groups from pure polymeric components, ceramics, and active ingredients, i.e., L-carnosine, fibroin, and sericin. In addition, during the 14-day incubation, a decrease in absorbance and a shift in peaks, as a result of the degradation of the ceramic–polymer coating, is noticeable on the FT-IR spectra of the c-C, c-F, and c-S samples. For the three samples tested, the most significant changes in FT-IR spectra were noticed after incubation in artificial saliva.

No significant differences due to the addition of sericin, fibroin, or L-carnosine were observed during incubation studies. The materials behaved similarly in the selected fluids. Changes in pH values confirmed the interaction with the incubation medium, which is also noticeable on the SEM images, as the effect is a change in surface morphology. Moreover, the changes in ionic conductivity indicate an interaction between the formed coatings and the fluid simulating the internal environment of the organism. All coatings in the examined liquids demonstrated sorption capacities with comparable values exceeding 200% over a 2-week period.

The antioxidant capacity of the *S. raeseri* extract was determined using the Folin–Ciocâlteu method and the DPPH free radical method. The study results indicate that the TPC for the aqueous extract of *Sideritis* is 31.64 ± 0.69 mg GAE/g. This value is similar to that obtained by Nikolay Yanchev et al., for whom, depending on the chosen solvent, it ranged from 4.0 to 32.2 mg GAE/g of dry plant material, while for the aqueous extract, it was 32.2 mg GAE/g of dry plant material [51]. Moreover, the DPPH test showed an inhibition percentage of 84.19 ± 0.71%, which is in line with the results obtained by Koleva et al. Depending on the *Sideritis* species used, the percentage of inhibition in hot water ranged from 78 to 88% [52]. Due to its high polyphenol content, *S. raeseri* extract was used to modify ceramic–polymer coatings on Ti-6Al-4V. A study of the release of polyphenols from the prepared coatings demonstrated a gradual release of the extract, which is associated with an increasing coefficient of the swelling capacity. The obtained material has the potential to be a carrier of active substances in a drug delivery system. In addition, the presence of an aqueous solution of the *Sideritis* extract increases the biological value of the obtained coating material. 

There is evidence of interactions between the samples and the incubation fluids, as shown in scanning electron microscopy images. The formation of new brushite crystals is influenced by Ringer’s fluid and PBS. After incubation, there were significantly more crystals in the samples than before, while incubation in water had little effect on crystal growth. This suggests that elements in Ringer’s fluid and PBS, such as calcium or phosphorus, activate the growth of brushite. And the growth of new layers may indicate the bioactivity of the material.

## 5. Conclusions

In the present study, flavonoid-modified ceramic–polymer materials were successfully prepared on a Ti-6Al-4V alloy. A method of brushite synthesis was proposed, and its physicochemical analysis was carried out. Moreover, the obtained coating materials were subjected to physicochemical analysis, incubation tests, and antioxidant tests of the active substance. Additionally, active ingredients derived from *S. raeseri* extract were released from the obtained coatings, which increased the biological value through the wide spectrum of applications for the employed plant. Using biodegradable materials for the coating and calcium phosphate ceramics, it is possible to apply the obtained active substance delivery system in bone tissue engineering and, in particular, in regenerative medicine. Based on the potential application of the obtained biomaterials, the results obtained suggest the importance of further research, in particular biological, cytotoxicity, and osteoconductive property studies.

## Figures and Tables

**Table 1:** Compilation of the composition of coating components.

Sample	15% PEG in *S. raeseri* Extract [mL]	L-Carnosine 2% [mL]	Fibroin 2% [mL]	Sericin 2% [mL]	Brushite [g]
c-C	5	5	-	-	0.175
c-F		-	5	-	
c-S		-	-	5	

## Data Availability

The data that support the findings of this study are contained within the article.
